# Perception of Discrete Emotions in Others: Evidence for Distinct Facial Mimicry Patterns

**DOI:** 10.1038/s41598-020-61563-5

**Published:** 2020-03-13

**Authors:** Tanja S. H. Wingenbach, Mark Brosnan, Monique C. Pfaltz, Peter Peyk, Chris Ashwin

**Affiliations:** 1 Centre for Applied Autism Research, Department of Psychology, University of Bath, Bath, UK; 2 Department of Consultation-Liaison Psychiatry and Psychosomatic Medicine, University Hospital Zurich, University of Zurich, Zurich, Switzerland

**Keywords:** Emotion, Physiology

## Abstract

Covert facial mimicry involves subtle facial muscle activation in observers when they perceive the facial emotional expressions of others. It remains uncertain whether prototypical facial features in emotional expressions are being covertly mimicked and also whether covert facial mimicry involves distinct facial muscle activation patterns across muscles per emotion category, or simply distinguishes positive versus negative valence in observed facial emotions. To test whether covert facial mimicry is emotion-specific, we measured facial electromyography (EMG) from five muscle sites (corrugator supercilii, levator labii, frontalis lateralis, depressor anguli oris, zygomaticus major) whilst participants watched videos of people expressing 9 different basic and complex emotions and a neutral expression. This study builds upon previous research by including a greater number of facial muscle measures and emotional expressions. It is the first study to investigate activation patterns across muscles during facial mimicry and to provide evidence for distinct patterns of facial muscle activation when viewing individual emotion categories, suggesting that facial mimicry is emotion-specific, rather than just valence-based.

## Introduction

Observing facial expressions of emotion in others often leads to congruent facial muscle activation in the observer that is not visible to the naked eye, which is termed covert facial mimicry. Facial electromyography (EMG) can be used to measure such subtle facial muscle activations due to its high sensitivity. Dimberg^1^ measured facial EMG activity from the zygomaticus major muscle (involved in smiling) and the corrugator supercilii muscle (involved in frowning) while participants passively viewed images of people facially expressing anger (includes frowning) and happiness (includes smiling). Participants were told that the study aimed at assessing physiological responses to faces and were instructed to pay attention to the faces. Participants were kept blind about the measurement of their facial muscle responses and thus told that their sweat response and heart rate were measured with the electrodes. Results showed that zygomaticus muscle activity in participants was greater while viewing happiness compared to anger expressions, while corrugator activity was greater in response to viewing anger compared to happiness expressions. Evidence for covert facial mimicry has since been reported across other emotion categories based on zygomaticus and corrugator muscle activity (e.g.^2–4^), showing that covert facial mimicry is evident in the activity of the corrugator and zygomaticus muscles of observers’ faces. Covert facial mimicry is further considered spontaneous and automatic, as it occurs without the individual’s awareness^5^.

However, the activity in the zygomaticus and corrugator muscles might simply reflect the valence of the observed emotion, with the zygomaticus muscle associated with positive valence and the corrugator with negative valence emotions^6–8^. This idea is consistent with findings from studies using affective stimuli showing that activity in the zygomaticus muscle is related to positive valence ratings, while corrugator muscle activity is associated with negative valence ratings^9^. Furthermore, increased corrugator muscle activity is reported during observation of different facial emotional expressions of negative valence, while increased zygomaticus activity is evident while watching facial expressions of positive valence^6,7,10–12^. The evidence using measures of corrugator and zygomaticus activity suggests facial muscles might code the valence of different emotional expressions in others. Such a valence-based differentiation fits well with the circumplex model of affect describing affect and also emotions as representable on the dimensions of valence (ranging from unpleasant to pleasant) and arousal (ranging from low to high in activity)^13^.

Though, other key emotion theories propose that there are specific facial expressions involving discrete facial actions for the discrete basic emotion categories of anger, disgust, fear, sadness, surprise, happiness, which are thought to have evolved due to adaptive functions they each serve^14–17^. Other emotions are often called complex emotions (e.g. pride), as they involve more complex cognitive processes such as self-evaluations^18^. The Facial Action Coding System (FACS)^19,20^ is an anatomical catalogue of all possible facial actions that are observable and details the corresponding facial muscle activation. One possibility for investigation of emotion-specificity of covert facial mimicry is to compare the measured facial muscle activation to the observed expression; specification of facial action and corresponding muscle can be determined by the FACS. A study measuring activity from the muscles corrugator, levator labii (wrinkles the nose), and lateralis frontalis (pulls the eyebrows up) tested for significant changes in response to facial expression of fear and disgust and found significant levator increase for disgust, a significant increase in corrugator activity for disgust and fear, and significant frontalis increase for fear^21^. These results show covert facial mimicry of the salient facial feature associated with these two basic emotion categories. However, knowledge on such emotion-specific covert facial mimicry for individual emotion categories is limited, as investigations thus far have mainly compared emotion categories to each other based on the activity of individual muscles.

Such studies have reported higher EMG activity in specific muscles of the face in observers while viewing others’ facial expressions of discrete target emotion categories compared to non-target emotion categories, including the levator muscle for disgust^10,17,18^, the corrugator muscle for sadness^22^, and frontalis muscle activity for fear^3,21^ and surprise^3,11,22^ as target emotion categories. Some studies failed to measure discernible covert facial mimicry activity entirely in certain muscles, particularly the frontalis and levator, when viewing others expressing fear and disgust respectively (see literature review by^6^). Künecke *et al*.^10^ examined the intensity of corrugator activation in response to viewing different facial emotional expressions and reported significant differences in the degree of corrugator muscle activity between the emotion categories. The literature thus shows some specific facial muscle activation when investigating covert facial mimicry but also overlap in activated muscles for some emotion categories. Combined with the fact that most published studies have only included measurements of the zygomaticus and corrugator muscles^6^, the current evidence for emotion-specific covert facial mimicry or the extent of it is limited. For example, there are no published reports on covert facial mimicry in response to viewing complex facial emotional expressions that we are aware of. It should be noted that discrete facial emotional expressions are brought about by the activation of multiple facial muscles. Investigations are needed assessing covert facial mimicry based on a wider range of facial muscle sites and examining the patterns of co-activation between them to test the valence-based versus discrete-emotion ideas of facial mimicry respectively the emotion-specificity of covert facial mimicry^11^.

**Figure 1:**
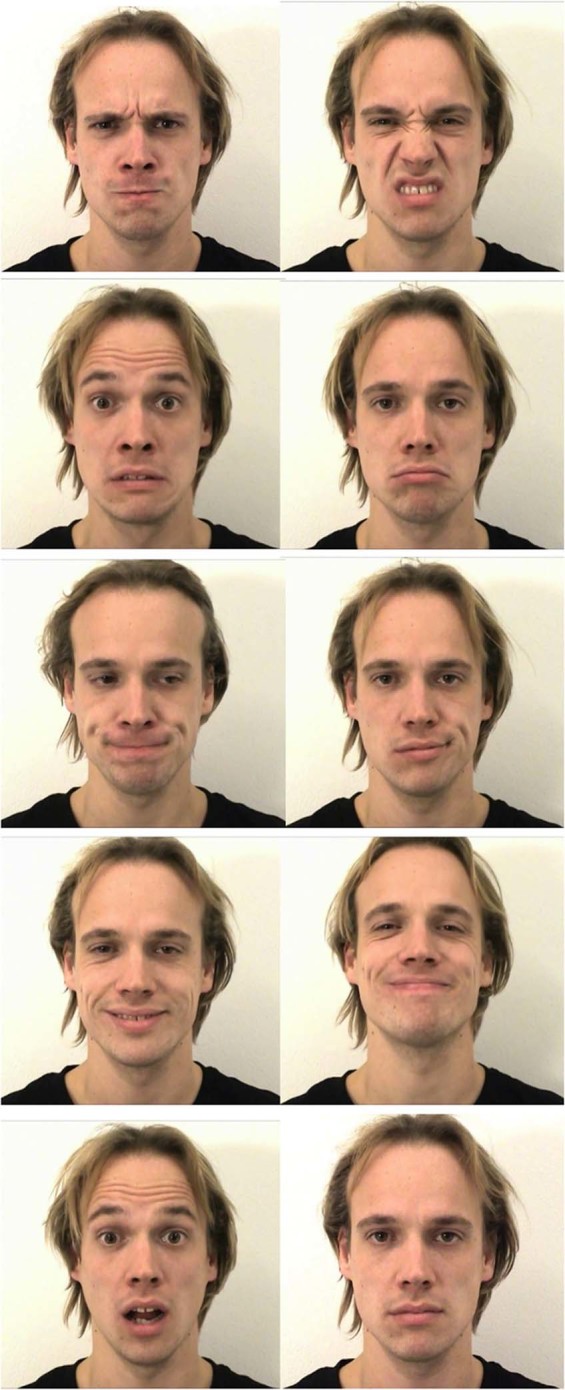
Example facial expressions for each of the nine emotion categories (from the top left corner per row: anger, disgust, fear, sadness, embarrassment, contempt, happiness, pride, surprise) and a neutral blank stare (last image bottom right corner) included in the study. The still images are the last frames of the high expression intensity video for each emotion category of the selected encoder.

In the present study, we measured facial EMG across five muscle sites while participants viewed videos of people expressing a range of different basic and complex emotions, as well as neutral expressions (see Fig. 1 for examples - the individual displayed in the image has given written informed consent to publish his photograph). The emotion categories included the positive valence expressions of happiness and pride, and the negative valence emotions included anger, disgust, fear, sadness, contempt, and embarrassment. We also included neutral expressions, which lack emotional valence, and the expression of surprise, which can contain both negative and positive valence aspects^23,24^. The aim was to investigate emotion-specificity of covert facial mimicry by examining similarity to prototypical facial emotional expressions and dissimilarity of the obtained facial mimicry patterns between the individual emotion categories. Regarding the similarity between covert facial mimicry and the stimuli, we hypothesised that individual facial muscle activation when observing facial emotional expressions would match the facial actions seen in the stimuli (see methods section for match-up per emotion category and Table 1). Regarding dissimilarity of covert facial mimicry between the individual emotion categories, we hypothesised that the facial muscle activation pattern associated with each observed emotion category would be distinct compared to at least one other facial muscle activation pattern within the valence category of the emotion tested.

**Table 1:** Emotion categories represented by facial action and allocated muscles and statistical contrast coefficients.

Emotion		Facial muscle site					
		Zygomaticus major	Depressor anguli oris	Levator labii	Corrugator supercilii	Frontalis lateralis	sum
anger	Facial actions			Lips tightened/pressed*	Brow lowered		
	coefficients	−2	−1	2	2	−1	0
disgust	Facial actions			Nose wrinkle			
	coefficients	1	−1	2	−1	−1	0
fear	Facial actions	Lips stretched			Brow lowered	Brow raised	
	coefficients	1	−1	−2	1	1	0
sadness	Facial actions		Lip corner dropped		Brow lowered	Brow raised	
	coefficients	−2	−2	−1	2	3	0
happiness	Facial actions	Lip corner pulled		cheek raised*			
	coefficients	2	−1	1	−1	−1	0
surprise	Facial actions		Jar dropped			Brow raised	
	coefficients	−1	−1	−1	−1	4	0
neutral	Facial actions						
	coefficients	1	1	−2	1	−1	0
contempt	Facial actions	Lip corner pulled				Brow raised	
	coefficients	2	−2	−1	−1	2	0
embarrassment	Facial actions	Lip corner pulled		Lips tightened/pressed*			
	coefficients	3	−1	1	−2	−1	0
pride	Facial actions	Lip corner pulled		cheek raised*			
	coefficients	2	1	1	−2	−2	0

*Note*. Contrast coefficients are based on expected increase (positive values) and reduction (negative values) in facial muscle activation in response to the facial emotion stimuli. Non-zero integers were chosen and the coefficients per emotion category needed to sum up to zero both for statistical purposes. *The facial actions are not produced by the levator labii, but the electrode pair placed in this facial region was assumed to record activity from these actions based on their proximity to the muscles producing the facial action.

## Results

### Similarity of muscle activation to stimulus categories

It was tested whether participants showed similar facial muscle activation to the movements shown in the stimuli for each emotion category. Table 2 presents the expected changes in facial muscle activity in response to the emotional expressions in the stimuli and the statistical results. Participants’ muscle responses to the emotional expression of *anger* did not match the activation of facial features in the stimuli, i.e. frowning and lip pressing, as the activity did not increase significantly in the corrugator site or the levator site respectively. The obtained facial muscle activation in response to the emotional expression of *disgust* matched the characteristic disgust expression of nose wrinkled, as the activity in the levator site increased significantly compared to baseline. The facial muscle activation in response to the emotional expression of *fear* partially matched the characteristic fear expression of stretched mouth corners (i.e. significantly increased activity in the zygomaticus site) but not raised and lowered eyebrows (i.e. no significant change in activity in the frontalis site and corrugator site respectively). The facial muscle activation in response to the emotional expression of *sadness* partially matched the characteristic sadness expression of pulling the eyebrows upwards (i.e. significantly increased activity in the frontalis site), but not of pulling the eyebrows together (i.e. no significant increase in activity in the corrugator site) and the lip corners dropping downwards (i.e. no significant decrease in activity in the depressor site). The facial muscle activation pattern in response to the emotional expression of *happiness* matched the characteristic happiness facial expression with mouth corners pulled outward and upward as the activity in the zygomaticus site increased significantly in response to happy facial expressions alongside a significant increase in levator site activity. The facial muscle activation pattern in response to the emotional expression of *surprise* matched the characteristic surprise expression, that is, dropping the jaw (i.e. significantly decreased activity in the depressor site) and raising the eyebrows (i.e. significantly increased activity in the frontalis site). The facial muscle activation in response to the emotional expression of *contempt* did not match the characteristic contempt expression of unilateral raised eyebrow and unilateral smile, as the activity did not significantly increase in the frontalis site and the zygomaticus site respectively. The facial muscle activation in response to the emotional expression of *embarrassment* partially matched the characteristic embarrassment expression of mouth corners pulling outward (i.e. increased activity in the zygomaticus site) but not lips pressed (i.e. no significant change in activity in the levator site). The facial muscle activation pattern in response to the emotional expression of *pride* matched the characteristic pride facial expression of mouth corners pulled outward and upward, as the activity in the zygomaticus site and in the levator increased significantly.

**Table 2:** Similarity of muscle activation to stimulus categories.

Emotion	Muscle	Hypothesis	*t*(72)	*p*	95% CI		Cohen’s *d*
anger	corrugator	↑	−1.31	0.098	−0.10	0.02	0.15
	levator	↑	1.44	0.098	−0.02	0.09	0.17
disgust	levator	↑ *	2.48	0.008	0.01	0.11	0.30
fear	zygomaticus	↑ *	2.31	0.018	0.01	0.09	0.25
	corrugator	↑ *	−4.24	<0.001	−0.16	−0.69	0.50
	frontalis	↑	−0.59	0.278	−0.05	0.03	0.07
sadness	corrugator	↑	0.57	0.385	−0.04	0.07	0.07
	frontalis	↑ *	3.37	<0.001	0.02	0.10	0.41
	depressor	↓	−0.29	0.385	−0.04	0.03	0.04
happiness	zygomaticus	↑ *	5.51	<0.001	0.10	0.22	0.64
	levator	↑ *	2.95	0.002	0.03	0.15	0.33
surprise	depressor	↓ *	−3.09	0.002	−0.13	−0.03	0.38
	frontalis	↑ *	3.37	<0.001	0.02	0.12	0.31
contempt	zygomaticus	↑	0.73	0.235	−0.03	0.06	0.08
	frontalis	↑	1.62	0.110	−0.01	0.07	0.19
embarrassment	zygomaticus	↑ *	2.00	0.050	0.00	0.07	0.23
	levator	↑	−0.72	0.238	−0.06	0.03	0.08
pride	zygomaticus	↑ *	7.61	<0.001	0.18	0.30	0.89
	levator	↑ *	3.88	<0.001	0.05	0.16	0.46

*Note*. Results from one sample *t*-tests for the expected changes in facial muscle activity in response to the facial emotional expression categories. ↑Expected increase in facial muscle activity. ↓Expected decrease in facial muscle activity. *Hypothesis confirmed. The presented *p*-values are adjusted for multiple comparisons and the significance level was set to 5%.

### Facial muscle activation patterns per emotion category

**Figure 2:**
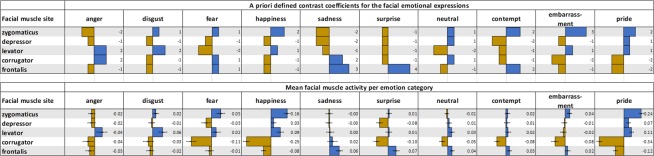
Expected and obtained facial muscle activation patterns per emotion category. Golden bars represent a reduction in activity in response to the stimuli and blue bars represent an increase in activity in response to the stimuli. Top panel: Expected patterns of facial muscle activation in response to the stimuli of each emotion category. A-priori defined contrast coefficients are represented numerically in the figure. Bottom panel: Baseline-corrected mean EMG activation of each facial muscle site in response to observing facial emotional expressions. Error bars represent the 95% confidence intervals of the means.

The observed EMG means for the five facial muscle sites measured per emotion category (see bottom panel of Fig. 2) were tested towards showing the expected facial muscle activation patterns (as represented by a-priori defined contrast coefficients; see method section and top panel of Fig. 2). Results showed that the EMG means in response to viewing facial expressions of the following emotion categories significantly fit the expected patterns: happiness, *t*(72) = 8.80, *p* < 0.001, *η*^2^ = 0.52; pride, *t*(72) =11.18, *p* < 0.001, *η*^2^ = 0.63; surprise, *t*(72) = 4.42, *p* < 0.001 *η*^2^ = 0.21; disgust, *t*(72) = 2.70, *p* = 0.008, *η*^2^ = 0.09; embarrassment, *t*(72) = 2.70, *p* = 0.008, *η*^2^ = 0.09; contempt, *t*(72) = 2.19, *p* = 0.023, *η*^2^ = 0.06, *η*^2^ = 0.63; sadness, *t*(72) = 1.90, *p* = 0.038, *η*^2^ = 0.05. The dissimilarity analyses comparing the facial muscle activation patterns between emotion categories of the same valence category to each other were thus conducted with the defined coefficient contrasts for these emotion categories.

The expected facial muscle activation pattern across the five muscles measured (i.e. contrast coefficient) in response to facial expression of anger, *t*(72) = 1.19, *p* = 0.119, *η*^2^ = 0.02, and fear *t*(72) = −1.31, *p* = 0.107, *η*^2^ = 0.02, were not found in the data. The expected facial muscle activation pattern in response to neutral facial expressions did also not appear in the observed data, *t*(72) = −2.68, *p* = 0.008, *η*^2^ = 0.09, as indicated by the significant negative *t*-value. Thus, additional exploratory analyses had to be conducted for the categories of anger, fear, and neutral; see section ‘Additional Analyses’.

### Dissimilarity analyses of positive valence emotions

**Figure 3:**
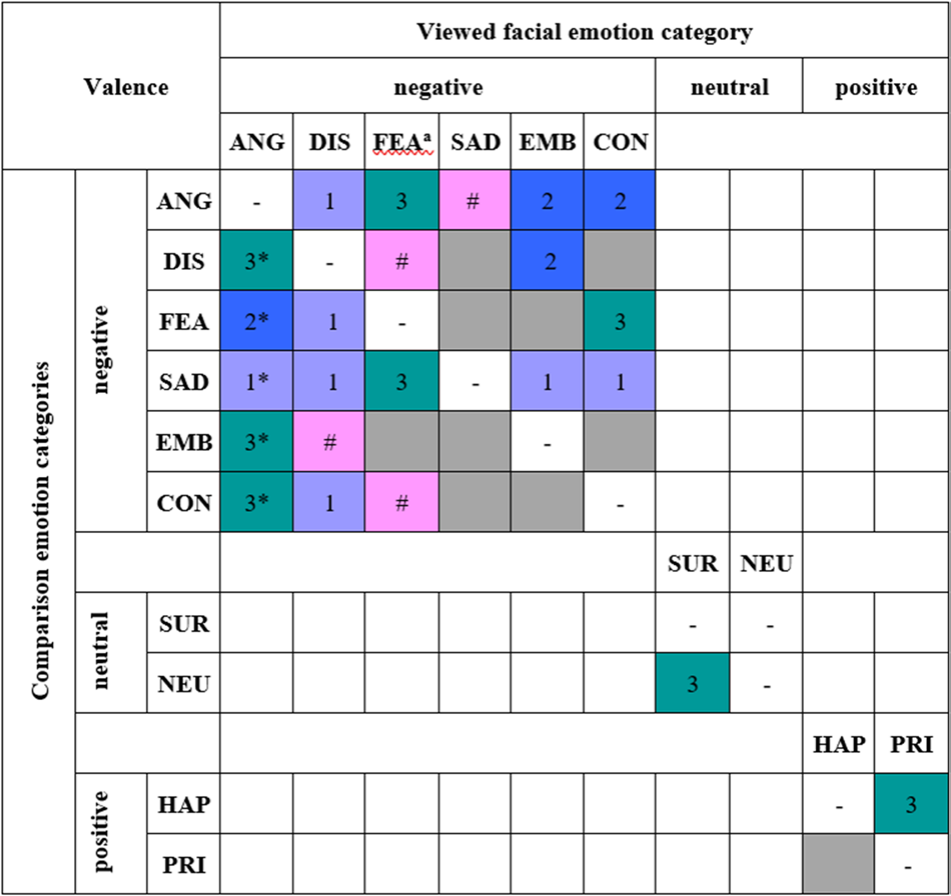
Facial muscle activation pattern contrast results per emotion category. The EMG data underlying the viewed emotion category and the associated expected facial muscle activation pattern were contrasted to the other expected patterns within the emotion’s valence category. ^**a**^For this emotion category, the facial muscle activation pattern was derived from the grand means of the observed data and contrasts conducted with this data-derived pattern. *The contrasts for the condition of viewing anger facial expressions were performed with *z*-standardised data. ANG = anger, DIS = disgust, FEA = fear, SAD = sadness, EMB = embarrassment, CON = contempt, SUR = surprise, NEU = neutral, HAP = happiness, PRI = pride. Grey rectangles represent non-significant results. 1 (purple) = *p* < 0.05, 2 (blue) = *p* < 0.01, 3 (green) = *p* < 0.001, **#** (pink) = *p* < 0.08.

The results from contrasting the EMG responses to viewing happiness facial expressions using the expected pattern of facial muscle activation for happiness to the expected pattern of pride (i.e. the other positive valence emotion) showed that the happiness pattern did not significantly better fit the data than the pride pattern, *t*(72) = −2.62, *p* = 0.006, *η*^2^ = 0.09. Whereas Fig. 2 displays the expected and obtained facial muscle activations across muscles for each emotion category, Fig. 3 shows a summary of the results from the dissimilarity analyses.

The results from contrasting the EMG responses to viewing *pride* facial expressions using the expected pattern of facial muscle activation for pride to the expected pattern of happiness (i.e. the other positive valence emotion) showed that the pride pattern did significantly better fit the data than the happiness pattern, *t*(72) = 4.05, *p* < 0.001, *η*^2^ = 0.19; see Fig. 3.

### Dissimilarity analysis of surprise

The results from contrasting the EMG responses to viewing *surprise* facial expressions using the expected pattern of facial muscle activation for surprise to the expected pattern of neutral showed that the surprise pattern did significantly better fit the data than the neutral pattern, *t*(72) = 5.75, *p* < 0.001, *η*^2^ = 0.31; see Fig. 3.

### Dissimilarity analyses of negative valence emotions

The EMG responses to viewing *disgust* facial expressions using the expected pattern of facial muscle activation for disgust were contrasted to the expected patterns of the other negative valence emotions; see Fig. 3. Results showed that the expected pattern of disgust significantly better represented the observed data than the expected pattern of anger, *t*(72) = 1.78, *p* = 0.049, *η*^2^ = 0.04; fear, *t*(72) = 2.59, *p* = 0.025, *η*^2^ = 0.09; sadness, *t*(72) = 2.28, *p* = 0.025, *η*^2^ = 0.07; and contempt, *t*(72) = 2.22, *p* = 0.025, *η*^2^ = 0.06. Compared to the expected pattern of embarrassment, the expected pattern of disgust trended towards significantly better describing the EMG data in response to disgust facial expressions, *t*(72) = 1.64, *p* = 0.053, *η*^2^ = 0.04.

The EMG responses to viewing *sadness* facial expressions using the expected pattern of facial muscle activation for sadness were contrasted to the expected patterns of the other negative valence emotions; see Fig. 3. Results showed that the expected pattern for sadness trended towards significantly better representing the observed data than the expected pattern of anger, *t*(72) = 2.35, *p* = 0.053, *η*^2^ = 0.07. The expected pattern of sadness did not significantly better fit the observed data than the patterns of disgust, *t*(72) = 1.53, *p* = 0.105, *η*^2^ = 0.03; fear, *t*(72) = 1.39, *p* = 0.105, *η*^2^ = 0.03; embarrassment, *t*(72) = 1.47, *p* = 0.105, *η*^2^ = 0.03; and contempt, *t*(72) = 0.76, *p* = 0.244, *η*^2^ = 0.01.

The EMG responses to viewing *embarrassment* facial expressions using the expected pattern of facial muscle activation for embarrassment were contrasted to the expected patterns of the other negative valence emotions; see Fig. 3. Results showed that the expected pattern of embarrassment significantly better represented the observed data than the expected pattern of anger, *t*(72) = 3.41, *p* = 0.003, *η*^2^ = 0.14; disgust, *t*(72) = 2.81, *p* = 0.009, *η*^2^ = 0.10; and sadness, *t*(72) = 1.98, *p* = 0.043, *η*^2^ = 0.05. The expected pattern for embarrassment did not significantly better describe the observed data than the expected pattern of fear, *t*(72) = 1.49, *p* = 0.088, *η*^2^ = 0.03, and contempt, *t*(72) = 0.06, *p* = 0.475, *η*^2^ = 0.00.

The EMG responses to viewing *contempt* facial expressions using the expected pattern of facial muscle activation for contempt were contrasted to the expected patterns of the other negative valence emotions; see Fig. 3. Results showed that the expected pattern for contempt significantly better represented the observed data than the expected pattern of fear, *t*(72) = 3.84, *p* < 0.001, *η*^2^ = 0.17; anger, *t*(72) = 2.83, *p* = 0.008, *η*^2^ = 0.10; and sadness, *t*(72) = 2.57, *p* = 0.010, *η*^2^ = 0.08. The expected pattern of contempt did not significantly better represent the observed data than the expected pattern of disgust, *t*(72) = 0.06, *p* = 0.476, *η*^2^ = 0.00, and embarrassment, *t*(72) = −0.44, *p* = 0.415, *η*^2^ = 0.00.

### Additional analyses for anger and fear

Additional exploratory analyses were conducted to determine whether there was a pattern of facial muscle activation in response to anger and fear facial expressions that merely diverged from the expected patterns, or there were no response patterns for these emotion categories at all. Thus, instead of a-priory defining contrast coefficients, contrast coefficients were now derived from the grand means per facial muscle site across participants. When the variance explained by the pattern is greater than the error variance in the sample, this indicates the existence of a significant response pattern in the data. A simulation analysis using synthetic random data was conducted to compare the type-I error level of a-priori defined contrasts to grand-average derived contrasts. The simulation data was generated to closely match our data specifications of 70 subjects per dataset, five multivariate normally distributed data channels with means of zero, and a covariance structure simulating the dependencies among EMG-channels (taken from our data). The rate of false positive results from 500,000 such data sets was 33% for grand-average derived contrasts and (the expected) 5% for a-priori defined contrasts; the obtained *p*-values for testing the contrasts for anger and fear for significance were thus multiplied by 6.63 (= 33/5). Since these contrast analyses were of exploratory nature, 2-tailed testing was applied^25^. Benjamini-Hochberg^26^ adjustment of the *p*-values was applied to account for multiple comparisons. Presented *p*-values are adjusted. Contrast analysis of the grand-average derived patterns showed that there was a significant pattern in the data in response to facial expressions of fear, *t*(72) = 5.42, *p* < 0.001, *η*^2^ = 0.29, but not in response to anger expressions, *t*(72) = 1.96, *p* = 0.358, *η*^2^ = 0.05. Dissimilarity analyses were conducted using the grand-average derived patterns for fear contrasted to the expected patterns of the other negative valence emotion categories.

Another simulation of the tests was conducted with random data (as described above) for grand-average derived contrasts when compared to a-priori defined contrasts and revealed a rate of false positive results of 13%. Thus, *p*-values were multiplied by 2.52 (=13/5). Since these contrast analyses were of exploratory nature, 2-tailed testing was applied^25^. Benjamini-Hochberg^26^ adjustment of the *p*-values was applied to account for multiple comparisons; presented *p*-values are adjusted. The results from contrasting the pattern in the data in response to facial emotional expressions of *fear* to the expected patterns of the other negative valence emotions showed that the fear pattern in the data was significantly different to the expected pattern of anger, *t*(72) = 5.17, *p* < 0.001, *η*^2^ = 0.27, and sadness, *t*(72) = 4.39, *p* < 0.001, *η*^2^ = 0.21, but not to the expected pattern of embarrassment, *t*(72) = 1.52, *p* = 0.338, *η*^2^ = 0.03; see Fig. 3. Results showed a trend towards a significant difference of the fear pattern to the expected pattern for disgust, *t*(72) = 2.45, *p* = 0.060, *η*^2^ = 0.08, and contempt, *t*(72) = 2.40, *p* = 0.060, *η*^2^ = 0.07.

### Further additional analyses for anger

**Figure 4:**
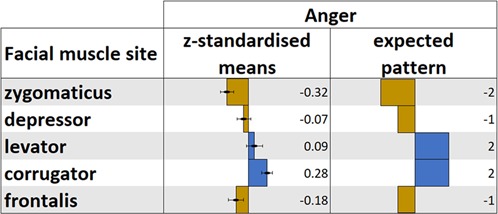
*Z*-standardised EMG responses to viewing facial expressions of anger and the expected facial muscle activation pattern.

The corrugator facial muscle site showed an unexpected reduction in activity in response to most stimuli categories (Fig. 2), as activity should have at least increased in response to facial emotional expressions of anger and fear based on published reports (e.g.^18^). It should be considered that corrugator activity is generally higher than many other muscles during tasks, as it also engages during attention even to simple sensory stimuli^27^. Given that the phenomenon investigated with the current study was covered facial mimicry an upper limit to which the corrugator activity could increase before becoming overt could be expected. In combination with participants having paid attention to the task, i.e. corrugator activation during baseline, it is conceivable that baseline-corrected responses did not show an increase in activity in response to anger facial expressions. Considering this occurrence, *z*-standardisation of the means was applied for each participant and separately for each facial muscle site across emotion categories. *Z*-standardisation allowed to investigate the facial muscle responses relative to the emotion categories included in the task. A figure displaying the *z*-standardised means of the five facial muscle sites per emotion category can be found in the Supplementary Fig. S1. The *z*-standardised facial muscle activation data for anger was then contrasted to the a-priori defined pattern for anger, tested 2-tailed based on the exploratory nature of this test^25^. Results showed that the *z*-standardised facial muscle activation in response to viewing anger facial expressions significantly fit the expected pattern, *t*(72) = 4.99, *p* < 0.001, *η*^2^ = 0.26; Fig. 4.

Further contrast analyses were conducted based on the *z*-standardised anger data to test the expected anger pattern against the a-priori defined patterns of the other negative valence emotions. Benjamini-Hochberg^26^ adjustment of the *p*-values was applied to account for multiple comparisons and *p*-values are presented as such; tests were conducted 2-tailed. Results showed that the pattern in the *z*-standardised anger data was significantly better described by the expected anger pattern than the expected pattern of embarrassment, *t*(72) = 4.59, *p* < 0.001, *η*^2^ = 0.23; contempt, *t*(72) = 4.86, *p* < 0.001, *η*^2^ = 0.25; disgust, *t*(72) = 3.80, *p* < 0.001, *η*^2^ = 0.17; fear, *t*(72) = 3.23, *p* = 0.003, *η*^2^ = 0.13; and sadness, *t*(72) = 2.07, *p* = 0.042, *η*^2^ = 0.06; Fig. 3.

## Discussion

We measured participants’ facial muscle activation in response to videos of others displaying facial expressions of basic and complex emotions to assess emotion-specificity of covert facial mimicry based on (1) similarity to the expression shown in the stimuli and (2) dissimilarity between the obtained facial muscle activation patterns. In line with the first hypothesis, measured facial muscle activations were largely congruent with emotion-specific facial actions displayed in the stimuli, demonstrating covert facial mimicry for most emotion categories included in the study. In line with our second hypothesis, the patterns of facial muscle activation across the five facial muscles measured were found to be distinct within the emotions’ valence categories, generally with moderate to large effect sizes. Together, the results from the current study show that covert facial mimicry is emotion-specific, rather than a reflection of an emotion’s valence.

Participants showed significant activations in response to the stimuli in expected muscle sites for 7 of the 9 emotion categories tested. Results revealed that covert facial mimicry of fear, embarrassment, happiness, and pride was found in the mouth region, covert facial mimicry of disgust was found around the nose, covert facial mimicry of sadness was found around the eye region and across the face for surprise. Since the facial expression of contempt includes unilateral facial muscle activation, it is possible that covert facial mimicry occurred in some participants on the facial site that was not measured and thus contributed to non-significant facial muscle activations for this emotion. The unexpectedly observed reduction in corrugator site activity in response to the stimuli can explain the lack of covert facial mimicry of anger expressions where the corrugator is underlying the main facial feature of this expression. Nonetheless, the results reported here show that there is substantial overlap between theoretically defined facial action per emotion category and the measured corresponding activity in individual muscles. Or in other words, the current results demonstrate similarity between observed facial actions and covert facial mimicry of these facial actions.

This similarity did not only apply to individual facial actions but also to the theory-derived and a-priori defined activation patterns across the five facial muscle sites, which were confirmed for all but two emotion categories (anger and fear) included in this study. It is particularly noteworthy that distinct patterns of covert facial mimicry were confirmed not only for basic emotions, but also for complex emotions. We believe that the current study is the first to report about covert facial mimicry of complex emotions, in addition to the basic emotions. The reduction in activation in the corrugator site in response to fear and anger expressions may have contributed to the lack of expected activation patterns for these two emotion categories. However, whereas exploratory analysis has not identified a pattern in the obtained EMG data in response to angry facial expressions, participants indeed presented a pattern of facial muscle activation in response to fearful facial expressions, albeit different from the expected pattern. Our results show that covert facial mimicry generally occurs in response to observing facial emotion, across both basic and complex emotion categories.

A possible explanation for the lack of covert facial mimicry of observed angry facial expressions could be that these are more threatening to the observer compared to the other emotion categories and that a mirroring of this expression could escalate a situation in a social interaction. This explanation is in line with the proposed function of *overt* mimicry to affiliate with others^6^. However, it should be noted that the current study investigated covert facial mimicry, i.e. facial muscle activation that was not visible and should therefore not have a negative effect on the other person. An alternative explanation is that the corrugator is a muscle that has a high basis activation due to its involvement in cognitive activities such as mental effort^28^. Visually inspecting EMG data from various studies conducted by us, we have observed a general increase in corrugator site activity in response to a stimulus, independent of the content of the stimulus. If the corrugator already shows higher activity before the target stimulus, then the response to the facial emotional expression during covert facial mimicry cannot easily become apparent when responses are baseline-corrected and given that there is an activation threshold under which covert facial mimicry has to remain before becoming overt. In consideration of this issue, we *z*-standardised the means to obtain a facial muscle activation pattern in response to viewing anger expressions relative to the other emotions viewed and the *z*-standardised means then fit the expected pattern for anger.

The expected patterns of facial muscle activations per emotion category matched the observed facial muscle activations for the same emotion category better than the expected patterns of other emotion categories of the same valence category. All negative valence emotion categories were found to better represent the expected pattern from at least one other negative valence emotion category. For example, the expected facial muscle activation pattern in response to disgust facial expressions better represented the observed data than the expected pattern of the negative valence emotions of anger, fear, sadness, and contempt, and trended towards significance compared to the expected pattern of embarrassment. The expected patterns for pride and surprise expressions, representing the positive and neutral valence category respectively, were also found to significantly better fit the observed facial muscle activation than the expected patterns of their comparison category. Although the expected pride and happiness patterns significantly fit the observed data of the respective emotion category, the expected pattern for happiness did not fit the facial muscle activation in response to happiness facial expressions better than the expected pride pattern. However, this is not surprising given the almost identical facial action underlying these two expressions and that the muscle lifting the chin (characteristic for pride) was not measured in the current study. These results show that the individual covert facial mimicry responses across the face are generally dissimilar to each other.

The pattern in response to anger based on the *z*-transformed means was significantly different to all other negative valence emotions included in the study. It is important to note that *z*-standardisation had differential effects on the data, i.e. some patterns of activity were unaffected by the transformation (e.g. pride, happiness), others were more aligned with the expected patterns (e.g. anger), and yet for other emotion categories the pattern disappeared altogether (e.g. fear); see Supplementary Fig. S1. Consequently, care needs to be taken when *z*-standardisation is applied to EMG data used for analyses and those results are subsequently interpreted.

Overall, our results are not consistent with ideas that covert facial mimicry merely reflects the valence of observed facial emotional^6–8^. Our findings are more consistent with theories defining emotions as discrete entities^14,29^ and with reports of a categorical structure of emotions based on their expression and experience, as well as their physiological and neural processes^30–32^. Noteworthy is a study where several discrete emotions were successfully identified based on neural activation patterns across multiple brain areas while participants imagined personal emotional experiences^33^. Though, we would like to emphasise that we do not consider covert facial mimicry itself to be an emotional response. Our results of emotion-specific facial mimicry patterns suggest that observed distinct emotional expressions are also perceived distinctively, at least on some level of the observers. The exact mechanisms underlying the differentiated covert facial mimicry response to others’ emotional expressions are not well understood to date.

That covert facial mimicry appears emotion-specific could be explained by embodiment theories of emotion (e.g. Niedenthal 2007) positing that the observation of facial emotional expressions can lead to a partial simulation of the observed emotion including its sensory and motor characteristics, based on an embodied neural representation of the emotion. In line with this, neuroscientific research showed that the emotion category of a perceived facial emotional expression can be predicted based on distinct neural activation patterns in the somatosensory cortex^34^. The somatosensory cortex has direct projections to the motor cortex and secondary projections to the premotor cortex^35^, which can explain the occurrence of covert facial mimicry. Whether emotion simulation following the observation of an emotional facial expression also includes changes in arousal should be addressed by future research beyond self-report by involving physiological measures such as electrodermal activity (alongside facial EMG).

As an alternative to the assumption that covert facial mimicry is linked to neural representation of emotions, it is possible that covert facial mimicry is not emotional in any sense and not even related to emotional content, but the result of perceiving movement per se. Mere motor simulation theories propose that motor simulation follows action observation^36^. There is evidence for equivalent brain activity in the observer and in the executer of an action from brain imaging studies with non-human primates showing activation of motor brain areas involved in executing hand and mouth movements in macaque monkeys observing these actions, but without visibly executing them^37–39^. Since facial emotional expressions are motor actions, covert facial mimicry could be based on the same mechanisms independent of the emotional component as if the facial expression was non-emotional. A neuroscientific literature review on social perception outlined that action observation recruits areas that process visual stimuli (e.g. PF, PFG), premotor cortex, and somatosensory cortex (particularly B2)^40^. That is, observing a facial emotional expression simulates the necessary motor output to execute the action oneself, which can explain the occurrence of covert facial mimicry; simulation of the somatosensory experience provides proprioceptive information for the involved facial actions. The activation of the neural representation of an emotion would not be necessary for this cascade. Like with other body movements linked to mirror neuron activation, covert facial mimicry could facilitate execution of the observed action either immediately (e.g. as a means of signalling) or in the future, related to training (e.g. clear facial communication)^41^. This explanation would suggest that covert facial mimicry only occurs when the observed action is sensible. Future research should include variations of facial expression (non-emotional facial expressions, mixed emotions, partial emotional facial expressions next to full emotional facial expression) to investigate whether covert facial mimicry is specific to emotions or a general occurrence to facial movement and whether partial emotional facial expressions are completed in the covert facial mimicry response.

There were some limitations of the current study. For example, the stimuli used had increased ecological validity based on the inclusion of expression intensity variations in video stimuli. But as a consequence, the emotion onset timings varied between the stimuli, which in turn led to varied timings in the onset of the facial muscle response to the stimuli. To most likely capture the facial responses to all stimuli, we had chosen a longer response window, which might have attenuated the mean facial responses. Thus, even clearer patterns of covert facial mimicry could be expected when video stimuli are used with standardised timings and shorter response windows for participants’ facial responses. The different intensities of emotional expression in the videos may also have modulated the facial responses in the participants. However, the effects of this factor were not tested in the current study, as it was not the focus of the research and the design did not have enough power based on the number of trials to include expression intensity as factor in the analyses. Whether expression intensity reflects within covert facial mimicry should be addressed by future research. Furthermore, participants were all university students within a limited age range, and so the results might not generalise to the wider population. Since covert facial mimicry is thought to be an automatic process, we predict the same results would be evident when tested in wider samples.

## Methods

### Participants

A total of 86 University students were recruited through campus advertising at the University of Bath from diverse departments. A required sample size estimation using G*Power^42^ to retrieve a small to medium effect revealed a total of 68 required participants with an alpha level of 0.05, power of 0.90 and an estimated effect size of 0.18. Technical equipment failure resulted in complete data loss for 6 participants, leading to a total of 80 participants (40 male, 40 female). The majority of the participants were Undergraduate students (*n* = 78) and 2 were Postgraduate students. Participants were 18 to 48 years old (*M* = 19.6, *SD* = 3.7), and all participants had normal or corrected-to-normal vision. Inspection of the EMG data for all 80 participants showed sustained signal corruption on one or more EMG channels for 7 participants; these participants were removed from any further data analyses. That is, 73 participants (38 male, 35 female; *M*(age) = 19.7, *SD* = 3.8) were included in the statistical analyses reported here. Ethical approval for the current study was granted by the University of Bath Psychology Research Ethics Committee and carried out in accordance with the provisions of the World Medical Association Declaration of Helsinki.

### Facial emotion stimuli

We used the validated Amsterdam Facial Expression Set – Bath Intensity Variations (ADFES-BIV^43^) as stimuli, which is an adaptation from the ADFES^44^. The video stimuli display a neutral facial expression that then develops into expressing either one of 9 emotions (anger, disgust, fear, sadness, surprise, happiness, contempt, embarrassment, pride; see Fig. 1) at three expression intensities (low, intermediate, high) or remains neutral. Twelve encoders (7 male, 5 female) express all emotions at each intensity level leading to a total of 360 videos in the stimulus set. Each video stimulus is 1040 ms in length.

Each video of the ADFES^44^ has been coded by certified coders^44^ according to the FACS^19,20^ assuring that the facial features most relevant for displays of individual emotional expressions are contained in the stimuli. A table presenting all coded facial actions for each emotion category included in the ADFES is presented in the respective publication (see^44^ and was used as basis for the pairing of facial muscle site to emotion category in the current study (Table 1). However, FACS-coding was not reported for neutral facial expressions, where only eyeblinks are included as facial movement.

### Facial mimicry task

E-Prime (Version 2.0^45^) was used as stimulus-presentation software. The study as a whole included all 360 videos of the ADFES-BIV^43^ but was divided in 4 parts each including 90 videos. The first part was to measure covert facial mimicry; the remaining 3 parts assessed facial emotion recognition (see^46^). Each part of the task included an equal distribution of encoders, emotion categories, and expression intensities. The videos in each part were presented in randomised order for each participant. Task instruction for the covert facial mimicry part was to passively watch the videos. Each trial started with a blank screen presented for 500 ms followed by a fixation cross in the centre of the screen for 500 ms, after which the stimulus video was presented. Following the stimulus video, there was a blank screen for 1500 ms as an ITI before the next trial started. The next trial started again with a blank screen for 500 ms (the change from blank screen to blank screen is not perceivable).

### EMG recording

The BIOPAC MP150 System with the Acqknowledge software (Version 4^47^) and EMG110C units for each of the five facial muscle sites (corrugator supercilii, zygomaticus major, levator labii, depressor anguli oris, and lateral frontalis) were used for recording of the EMG data with a gain of 2000. Pairs of shielded surface silver-silver chloride (Ag-AgCl) electrodes (EL254S) filled with conductive gel (saline based Signa Gel) and with a contact area of 4 mm diameter were used. The EMG signal was bandpass filtered online from 10 Hz to 500 Hz. Grounding was achieved through the VIN- of the TSD203, GSR (the data from this was not for research purposes). The sampling rate was 1000 Hz throughout the experiment.

### Procedure

Participants were tested individually in a quiet laboratory at the University of Bath with the experimenter present. Written informed consent was obtained prior to study participation. Participants were seated approximately 60 cm from the PC monitor (17”) with a screen resolution of 1024 × 768 pixel, matching the resolution of the ADFES-BIV^43^ videos. The faces thus appeared approximately life-sized to the participants, similar to face-to-face conversations. Participants’ faces were cleaned with alcohol swabs to remove any oil or make-up from the face to help secure electrode attachment of the double-stick adhesive rings to the skin. The 10 facial EMG electrodes were then placed in pairs over the respective muscle sites on the left side of the face according to the guidelines by Fridlund and Cacioppo^48^. The electrodes of each pair were placed in close proximity to each other with a distance of approximately 1 cm between the electrode centres. The lower electrode on the lateral frontalis was placed in line with the pupil of the eye when looking straight ahead approximately 1 cm above the eyebrow, and the second electrode was placed just above. For the corrugator supercilii, one electrode was placed directly above the eyebrow lined up with the inner commissure of the eye fissure, and the other electrode was placed lateral and slightly above the first one above the eyebrow. For assessment of the levator labii, one electrode was placed lateral to the baseline of the nostril, and one electrode right below and slightly lateral of the first one. To assess zygomaticus major activity, one electrode was placed in between the corner of the mouth and the bony dimple next to the ear, and one electrode toward the mouth on the same imaginary line. The first electrode on the depressor anguli oris was placed about 1 cm below the corner of the mouth and the second was placed below the first one. Participants were kept blind about the true purpose of the study of assessing their facial muscle activity when watching others’ facial emotion. Thus, participants were told that the electrodes would be measuring pulse and sweat response to facial emotional expressions akin to Dimberg^1^. Participants were further asked to sit still for optimal assessment of their body’s responses.

The task started with a short neutral-content clip (4 min 18 sec), which was included to induce a similar neutral affective state in all participants to begin the experiment^43^. Participants then underwent the facial mimicry task reported in this manuscript (see section Facial Mimicry Task), followed by a facial emotion recognition task which is presented elsewhere^46^. After completion of the computer-task, participants were debriefed and compensated with either course credit or GBP 7.

### Data processing and preparation

The Autonomic Nervous System Laboratory 2.6 (ANSLAB^49^) was used for post-processing of the EMG data. For each recording site of every participant, the recorded EMG signal was 50 Hz notch filtered, 28 Hz high-pass filtered, rectified and smoothed with a moving average width of 50 ms. EMG signals were then down-sampled to 100 Hz. Covert facial mimicry has been reported to be discernible after approximately 400 ms after facial emotional expression onset^4^ and to reach its maximum within 1 second^2^. It should be noted that there are considerable variations in emotion onsets in the video stimuli used in the current study (for more detail, see^43^). Thus, it is possible that for some videos the emotional information became visible late in the video (e.g. for low intensity facial emotional expressions). For example, if emotion onset in one video was 800 ms after stimulus onset and the maximum facial mimicry response can be expected around 1000 ms after emotion onset, then the response window for calculating the mean EMG activation should include 1800 ms after stimulus onset to capture the maximum response. Consequently, a larger response window was chosen in the current study than in studies with standardised emotion onsets in the stimuli. The down-sampled EMG signal of each recording site was segmented into individual trials with a timing period starting from 500 ms before stimulus onset until 2000 ms after stimulus onset. The pre-stimulus baseline was defined as the 500 ms before stimulus onset and the response window was defined as starting 700 ms until 2000 ms after stimulus onset.

Trial data were down-sampled further to 10 Hz, but only for the detection of artefactual spikes (for all other analysis steps described below the 100 Hz-sampled trials were used). Trials were excluded if the difference between two adjacent data points was greater than 3 SD of the entire trial. The remaining trials were then submitted to an identification step of improbable data which was performed using EEGLAB functions^50^. Trials were excluded if their summed sample probability deviated from the mean probability of all trials of each channel by more than 3 SD. The remaining number of trials (spike-filtered and probability-edited) per EMG channel and emotion category ranged from 6 to 9, and did not differ significantly between emotion categories for any of the 5 EMG channels (*F*’s(9, 711) < 1.50, *p*’s > 0.142). Finally, the variability of the data was normalised within each participant and facial muscle site to compensate for inter-individual and inter-muscle anatomical differences. That is, each data point for a participant within an EMG channel was divided by the SD of all sample points of the respective EMG channel in the remaining trials. The applied formula assured comparability of the data variance across subjects and channels while conserving the interpretability of the data after baseline-correction (i.e. increase/decrease of facial muscle activity in response to the stimuli), unlike with *z*-standardisation.

As last step of data preparation for statistical analysis, means for EMG activation in response to the stimuli were calculated per trial over the duration of the specified response window (i.e. 700–2000 ms after stimulus onset). The trial means were averaged for each participant by emotion category (10) and muscle (5), resulting in a total of 50 means for each participant.

### Statistical analyses

To test for similarity of the observed facial muscle activations to the facial emotional expressions displayed in the stimuli, one sample *t*-tests were conducted. Since the EMG data were baseline-corrected per stimulus, the resulting means can be tested against 0 to reflect an increase in activity in response to the stimulus or a decrease in activity in response to the stimulus. In accordance with Table 1, the following tests were conducted per emotion category: anger = corrugator (+) and levator (+); disgust = levator (+); fear = zygomaticus (+), corrugator (+), and frontalis (+); sadness = corrugator (+), frontalis (+), and depressor (−); happiness = zygomaticus (+) and levator (+); surprise = depressor (−) and frontalis (+); contempt = zygomaticus (+) and frontalis (+); embarrassment = zygomaticus (+) and levator (+); pride = zygomaticus (+) and levator (+). The plus and minus signs in the parentheses indicate the expected direction of the effect. It should be noted that facial muscle *sites* rather than specific individual facial muscles were assessed, meaning that some activity could also be expected to be recorded by an electrode site when the target movement was carried out by a different muscle. No tests were conducted for the neutral stimuli, as (besides eye blinks) no facial movement was visible in the experimental stimuli. All tests were conducted against a significance level of 5% (1-tailed) in SPSS (Version 25^51^). Benjamini-Hochberg^26^ adjustment of the *p*-values within conditions (i.e. emotion category) was applied to account for multiple comparisons and the presented *p*-values were adjusted. Cohen’s *d* was calculated (*d* = mean difference/SD) and is presented as effect size measure; 95% CIs for the mean differences are presented.

To test whether the obtained EMG patterns in response to passively viewing facial emotional expressions were distinct, a 2-step process of analysis was applied using contrast analysis. Contrast analysis allows to test theory-driven a-priori predictions where contrast weights are generated reflecting the hypothesised pattern of means in the data^25^. In the case of pure repeated-measures design, the test can be considered a generalisation of the *t*-test for dependent samples, and includes the calculation of the covariance of each participant’s data with the contrast coefficients (‘*L*-scores’); the average of these covariances can then be *t*-tested against zero (see^25^). Thus, *L*-scores reflect the degree to which a participant’s data represents the expected pattern. In our case, zero represented a lack of facial muscle activation in response to the stimuli. Consequently, significant positive *t*-values show that the observed data is represented well by the expected pattern (whereas a significant negative *t*-value would indicate the opposite of the expected pattern in the data).

Contrast coefficients were a-priori defined for each of the 9 emotion categories included in the current study as expected for prototypical facial emotional expressions (Table 1). The contrast coefficients per emotion category were required to be centred (i.e. sum up to 0) and to comprise of non-zero integers only. The coefficients were defined in compliance with these criteria while considering the coded facial action for the stimuli presented in Table 1. Positive coefficients represent an assumed increase in facial muscle activity and negative coefficients represent an assumed decrease in facial muscle activity in response to the stimuli. A contrast coefficient was also defined for the category of neutral facial expressions (as comparison category for surprise) and had to comply with the same specifications as for the emotional facial expressions. Thus, positive coefficients were chosen for facial muscle sites associated with the emotion categories with which confusions of neutral expressions can occur within facial emotion recognition tasks (e.g.^43,52^), i.e. anger and corrugator, sadness and depressor, happiness and zygomaticus.

The first analysis step then was to calculate the degree to which each participant displayed the expected pattern of EMG activation per emotion category (i.e. *L*-scores) and to test the observed sample means for significant representation of the expected patterns. To exemplify, it was tested whether participants’ EMG responses to observed happiness facial expressions matched the expected facial muscle activation pattern of happiness (i.e. the defined contrast for happiness); this was done for all 10 emotion categories. These tests were implemented in a custom-made script in Matlab (Version 2019a^53^). Since confirmatory contrast analysis was applied, which is inherently directional^25^, *t*-tests were conducted 1-tailed against an alpha level of 5%; eta squared was derived from the *t*-statistics and used as effect size measure. However, Benjamini-Hochberg^26^ adjustment of the *p*-values was applied to the 10 tests to account for multiple comparisons and the adjusted *p*-values are presented in the results. Significant test results from this step were considered a prerequisite for the second analysis step.

In a second step, the facial muscle activation patterns per emotion category were tested for dissimilarity within their respective valence category in pairs, again using contrast analysis following the suggestion by Furr and Rosenthal^25^. This time, the contrast coefficients per emotion category were first *z*-transformed to make them comparable among each other. Then, contrast analysis was conducted using the *z*-transformed coefficient vectors of the two patterns to be compared. It was tested whether the observed EMG means in response to facial emotional expressions of a specific category better described the expected contrast pattern of this emotion category or the comparison emotion’s expected contrast pattern. The following comparisons were planned for the negative valence category: anger – disgust; disgust – anger; anger – fear; fear – anger; anger – sadness; sadness – anger; anger – contempt; contempt – anger; anger – embarrassment; embarrassment – anger; disgust – fear; fear – disgust; disgust – sadness; sadness – disgust; disgust – contempt; contempt – disgust; disgust – embarrassment; embarrassment – disgust; fear – sadness; sadness – fear; sadness – contempt; contempt – sadness; sadness – embarrassment; embarrassment – sadness; contempt – embarrassment; embarrassment – contempt. The following comparisons were planned for the positive valence category: happiness – pride; pride – happiness. The following comparison was planned for the neutral valence category: surprise – neutral.

It should be noted that the emotion category pairs had to be tested bi-directionally, because the data underlying the first listed emotion category was always contrasted to the expected pattern of the first and second listed emotion category; results could thus differ between tests of the same emotion pair. To exemplify, it was tested whether the facial muscle activation means in response to happiness facial expressions better matched the expected pattern of happiness or the expected pattern of pride; and then it was tested whether the EMG data in response to pride facial expressions better matched the expected pattern of pride or the expected pattern of happiness. Thus, each participant’s *L*-scores were calculated for both emotion contrasts to be compared and the difference between the two *L*-scores was computed. Consequently, positive *L-*scores indicated the participant displayed the pattern of the first emotion’s expected pattern more than the expected pattern of the second emotion; vice versa for negative *L*-scores^25^. The sample difference scores were then submitted to significance testing; significance indicated the degree to which the observed data better represented the target emotion’s expected pattern than the comparison emotion’s expected pattern. Since confirmatory contrast analysis was applied, which is inherently directional^25^, *t*-tests were thus conducted 1-tailed against an alpha level of 5% and eta squared was derived from the corresponding *t*-statistics as effect size measure. Benjamini-Hochberg^26^ adjustment of the *p*-values was applied to account for multiple comparisons for each emotion category, e.g. 5 comparisons were conducted for disgust and thus the adjustment was applied to those five tests. These tests were also implemented in Matlab (Version 2019a^53^).

## Supplementary information

Supplementary information.

## Data Availability

All data underlying the analyses reported here are freely accessible from the open data repository OSF (10.17605/OSF.IO/MUKDZ).
