# Supplementary material for: Perception of Discrete Emotions in Others: Evidence for Distinct Facial Mimicry Patterns
Source: Sci Rep. 2020 Mar 13;10:4692. doi: 10.1038/s41598-020-61563-5 (PMC7069962; doi:10.1038/s41598-020-61563-5)
Supplement: Supplementary file 1 — Supplementary information. [file 41598_2020_61563_MOESM1_ESM.pdf]

Perception of Discrete Emotions in Others: Evidence for Distinct Facial Mimicry Patterns

Tanja S. H. Wingenbach, Mark Brosnan, Monique C. Pfaltz, Peter Peyk, Chris Ashwin

Supplementary Figure S1

z -standardised means	***Z*** **-standardised mean facial muscle activity per emotion category**									
**Facial muscle site**	**anger**	**disgust**	**fear**	**happiness**	**sadness**	**surprise**	**neutral**	**contempt**	**embarrass-ment**	**pride**
**zygomaticus**	-0.32	-0.15	-0.02	0.48	-0.20	-0.20	-0.21	-0.15	-0.11	0.89
**depressor**	-0.07	0.04	-0.17	0.18	-0.08	-0.37	0.12	0.10	-0.07	0.32
**levator**	0.09	0.13	-0.12	0.26	-0.17	-0.14	0.00	-0.07	-0.34	0.35
**corrugator**	0.28	0.34	-0.02	-0.63	0.58	-0.01	0.26	0.08	0.18	-1.04
**frontalis**	-0.18	-0.10	-0.09	-0.38	0.40	0.35	0.31	0.26	0.12	-0.69

Z-standardised facial muscle activation patterns per emotion category. Standardisation was conducted on the mean responses for each participant and EMG channels separately. Golden bars represent lower than average activity in response to the stimuli and blue bars represent greater than average activity in response to the stimuli. Error bars represent the 95% confidence intervals of the means.
